# LINE-1 ORF1p expression occurs in clear cell ovarian carcinoma precursors and is a candidate blood biomarker

**DOI:** 10.1038/s41698-025-00849-1

**Published:** 2025-03-06

**Authors:** Pamela R. de Santiago, Sho Sato, Stephanie J. Zhang, Meaghan C. Dougher, Kyle M. Devins, Agnes J. Bilecz, Sagar Rayamajhi, Gabriel Mingo, Hannah S. Rendulich, Yi Feng, Connie Wu, Martin S. Taylor, Yelena Zhuravlev, Euihye Jung, Dalia K. Omran, Tian-Li Wang, Ie-Ming Shih, Lauren E. Schwartz, Sarah Kim, Mark A. Morgan, Janos L. Tanyi, Kathleen H. Burns, Ernst Lengyel, Carlos Parra-Herran, Andrew K. Godwin, David R. Walt, Ronny Drapkin

**Affiliations:** 1 Penn Ovarian Cancer Research Center, Perelman School of Medicine, University of Pennsylvania, Philadelphia, PA USA; 2 Department of Pathology, Brigham and Women’s Hospital and Harvard Medical School, Boston, MA USA; 3 Wyss Institute for Biologically Inspired Engineering at Harvard University, Boston, MA USA; 4 Department of Pathology, Hospital of the University of Pennsylvania, Philadelphia, PA USA; 5 Department of Obstetrics and Gynecology/Section of Gynecologic Oncology, University of Chicago, Chicago, IL USA; 6 Department of Pathology and Laboratory Medicine, University of Kansas Medical Center, Kansas City, KS USA; 7 Department of Pathology, Massachusetts General Hospital and Harvard Medical School, Boston, MA USA; 8 Johns Hopkins University School of Medicine, Baltimore, MD USA; 9 Department of Obstetrics and Gynecology, Division of Gynecologic Oncology, Hospital of the University of Pennsylvania, Philadelphia, PA USA; 10 Department of Pathology, Dana Farber Cancer Institute and Harvard Medical School, Boston, MA USA; 11 Kansas Institute for Precision Medicine, University of Kansas Medical Center, Kansas City, KS USA; 12 Basser Center for BRCA, Abramson Cancer Center, Perelman School of Medicine, University of Pennsylvania, Philadelphia, PA 19104 USA

**Keywords:** Ovarian cancer, Ovarian cancer

## Abstract

Long interspersed element 1 (LINE-1) retrotransposons are repetitive sequences that can move within the genome by an autonomous mechanism. To limit their mutagenic potential, benign cells restrict LINE-1 expression through molecular mechanisms such as DNA methylation and histone modification, but these mechanisms are usually impaired in cancer. Clear cell ovarian carcinoma (CCOC) represents 5–10% of ovarian cancers and is thought to arise from endometriosis. Women with advanced CCOC face poor prognoses, highlighting the importance of understanding early disease pathogenesis. In our study, 33 of 40 cases (over 82%) of CCOC tumors express ORF1p, a LINE-1-encoded protein. We found that LINE-1 de-repression is an early event in CCOC, as ORF1p is enhanced during the transition from typical to atypical endometriosis and persists in invasive cancer. Finally, using single-molecule array (Simoa) assays, we detected ORF1p in patient blood, suggesting it as a potential minimally invasive biomarker for this disease.

## Introduction

Mobile genetic elements make up nearly half of the human genome and can be grouped into two major classes: DNA transposons and retrotransposons. In humans, retrotransposons are most prevalent and currently active. This activity is fueled by long interspersed element 1 (LINE-1, L1) retrotransposons. With about 500,000 copies, LINE-1 interspersed repeats constitute approximately 17% of the human genome. About one hundred LINE-1 elements are potentially active today in any human genome^1–3^. To counteract the potentially deleterious effects of mobile element insertions, host mechanisms have evolved to combat retrotransposition. For instance, the transcription of LINE-1 is driven by a CpG dinucleotide-rich internal promoter, and expression in adult human cells is usually suppressed by molecular mechanisms including DNA methylation and transcriptional regulation^4–6^. Post-transcriptional control mechanisms have also been described^7–9^. Interestingly, a number of studies have documented the aberrant expression of LINE-1 across epithelial cancers, including ovarian cancer^10–15^. In most cases, DNA hypomethylation is associated with LINE-1 reactivation^10–13,16,17^.

Epithelial ovarian cancer is a heterogeneous disease with numerous histopathologic and genetic types. The most common histopathologic variants include high-grade serous, endometrioid, clear cell, mucinous, and low-grade serous carcinomas. High-grade serous ovarian carcinoma (HGSOC) is the most common subtype, accounting for up to 70% of cases and the majority of disease-related mortality^18^. Clear cell ovarian carcinoma (CCOC) is less common, accounting for 5–10% of ovarian cancer in North America, although it is more common in Asia^19^. CCOC and endometrioid ovarian carcinomas demonstrate similar driver mutations and are both thought to derive from endometriosis^20–23^. Thus, they are sometimes referenced together as endometriosis-associated ovarian carcinomas.

The majority of CCOC is diagnosed at early stage with an overall favorable prognosis. However, CCOC is notoriously challenging to treat when detected at an advanced stage. In such case, it exhibits a markedly low response rate to standard chemotherapeutic regimens and is associated with poor prognosis^24^. There are currently no effective therapies for women with advanced CCOC, and a major obstacle in improving the outcomes for patients is the incomplete understanding of its pathogenesis. Specifically, there is a lack of comprehensive understanding of the factors driving its origin from endometriosis, its aggressive tumor behavior, and its poor response to therapies^25^.

Here, we investigate the expression of LINE-1 in CCOC and precursor endometriotic lesions. We found that the majority of these tumors express ORF1p, one of two open reading frames (ORFs) encoded by LINE-1. Due to the association of clear cell ovarian carcinoma and endometriosis, we examined whether ORF1p expression is detectable in typical and atypical endometriosis. By immunohistochemistry, we found that the transition from typical endometriosis to clear cell carcinoma is marked by enhanced ORF1p expression, which is retained in invasive disease. We show that CCOC cell lines retain the expression of ORF1p and find accumulation of ORF1p in extracellular media. The release of ORF1p into conditioned media is explained, in part, by its presence in small extracellular vesicles (sEVs). Finally, we employed single-molecule array (Simoa) assays to detect ORF1p in the blood samples of patients with CCOC. Our findings reveal that ORF1p is consistently expressed across serum and plasma samples, particularly in patients at the early stage (Stage I) of CCOC, highlighting its promise for early diagnosis in CCOC cases. Together, these results suggest that de-repression of LINE-1 elements is an early event in clear cell ovarian carcinomas and that ORF1p could serve as a diagnostic biomarker for this disease.

## Results

### LINE-1 is de-repressed in clear cell ovarian carcinomas

**Fig. 1:**
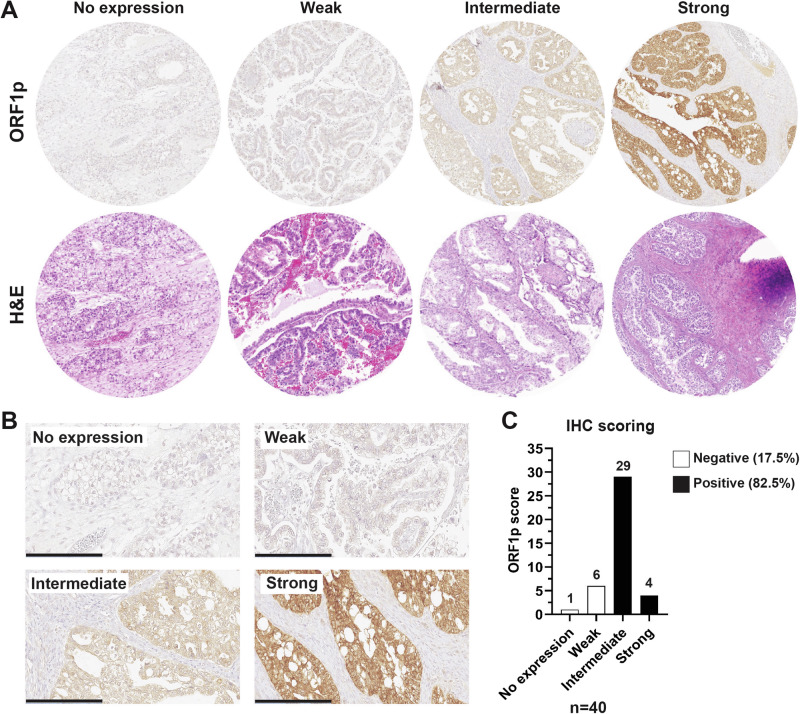
LINE-1 is de-repressed in clear cell ovarian carcinoma. **A** Formalin-fixed, paraffin-embedded human tissue from TMA. Representative H&E (bottom panel) and ORF1p IHC (upper panel) scale images used for scoring. **B** ORF1p IHC scale at higher magnification. Scale bar: 200 µm. **C** Distribution of ORF1p scoring in CCOC samples (*n* = 40).

LINE-1 deregulation and concomitant expression of open reading frame 1 protein (ORF1p) is a common feature of many cancer types, including ovarian carcinoma^6,13,26^. The observation that high-grade serous carcinomas, the most common subtype of ovarian cancer, express ORF1p prompted us to investigate whether other subtypes also express this protein^11,13,14^. In particular, we were interested in clear cell ovarian carcinomas (CCOC) because they represent a challenging histotype to treat if detected at late stage. We created a tissue microarray (TMA) of forty CCOC cases and used immunohistochemistry (IHC) to assess ORF1p expression. ORF1p staining was scored by five anatomic pathologists using a 4-tiered scale (no expression, weak, intermediate, and strong) (see Methods, LINE-1 ORF1p Immunohistochemistry scoring) (Fig. 1A). Results showed that staining was restricted to the epithelial cells, with no staining in the stromal compartment. A fine speckled cytoplasmic pattern of staining was noted in all positive cases, consistent with previous reports^13–15^ (Fig. 1B). In cancer samples, ORF1p expression was generally robust and diffuse (72.5% intermediate, 10% strong), though immunoreactivity varied between cases. When we dichotomized the scores into negative (no expression and weak) and positive (intermediate and strong), we observed that 33 out of 40 (82.5%) of the CCOC cases were positive (Fig. 1C), together demonstrating that LINE-1 is pervasively de-repressed in CCOC.

To assess the stability of ORF1p as a marker, we also evaluated two cases of recurrent disease. As is shown in Supplementary. Fig. 1, ORF1p showed a strong expression in pre- and post-chemotherapy treatment samples from both cases, suggesting that the stability of ORF1p could also be used in patients with recurrent disease.

Finally, we asked whether there is a correlation between ORF1p tumor expression and clinical variables. We did not detect any correlation with age at diagnosis (*p*-value = 0.55), FIGO stages *p*-value > 0.99), or tumor size (*p*-value = 0.35) (Supplementary Table 3). Together with the diffuse and homogeneous staining pattern across tumor samples, we suggest that LINE-1 expression levels, as measured by ORF1p, may reach a ‘threshold’ in carcinogenesis that is consistent through late stages of progression.

### ORF1p expression is enhanced during neoplastic transformation

**Fig. 2:**
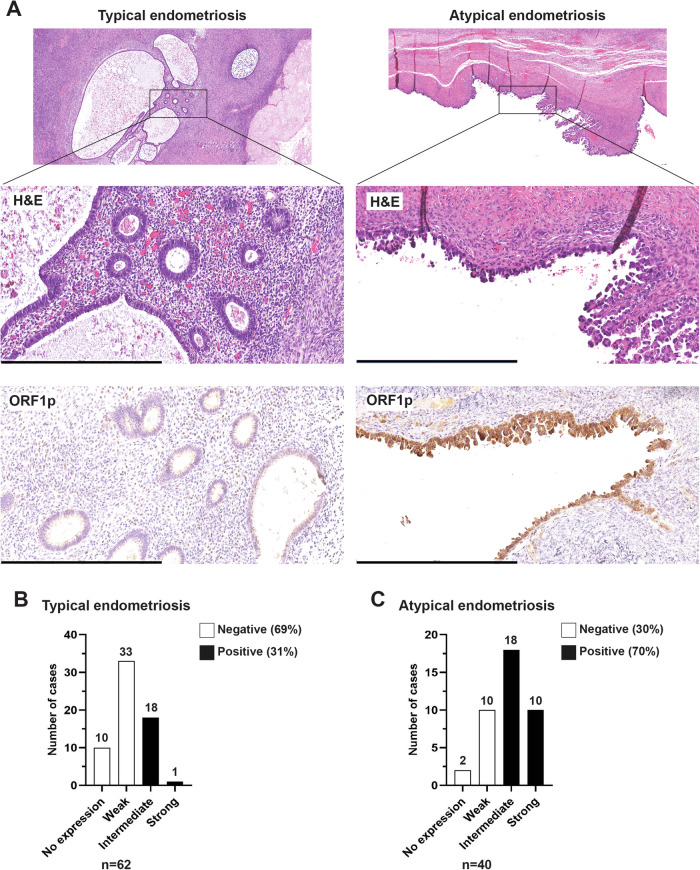
ORF1p expression is enhanced during neoplastic transformation. **A** Representative images of hematoxylin and eosin (H&E) staining and ORF1p expression (IHC) on whole-mount slides from cases of typical endometriosis (left panel) and atypical endometriosis (right panel). Scale bar: 500 µm. **B** ORF1p IHC scoring for typical endometriosis (*n* = 62) and **C** atypical endometriosis (*n* = 40).

The observation that CCOC samples express ORF1p motivated us to ask whether its de-repression is an early event in the pathogenesis of this disease. The link between endometriosis and CCOC is well established^21–23,27,28^. Temporal acquisition of genetic and epigenetic alterations in endometriotic lesions (typical endometriosis) leads to morphologically visible epithelial atypia (atypical endometriosis) and eventual carcinoma^29^. To investigate whether LINE-1 expression occurs in these early lesions, we performed ORF1p IHC on whole-mount slides from cases of typical endometriosis (*N* = 62) and atypical endometriosis (*N* = 40). As observed in CCOC samples, ORF1p staining was restricted to the epithelial lining, and was negative in the endometrial stroma (Fig. 2A). Positive staining of typical endometriosis samples was noted in 19 out of 62 (31%). Interestingly, we observed a much higher frequency of expression in atypical endometriosis samples, where positive staining for ORF1p was found in 28 out of 40 cases (70%) (Fig. 2B). As a control, we evaluated ORF1p expression in 60 benign proliferative (*n* = 35) and secretory (*n* = 25) endometrium samples, which showed negative staining in the majority of the cases (66% and 76%, respectively) (Supplementary Fig. 2). The increased immunoreactivity observed in atypical vs. typical endometriotic lesions (Supplementary Table 4, *p*-value = 0.0001) and benign endometrium, suggests that ORF1p expression is enhanced during neoplastic transformation.

### LINE-1 ORF1p is expressed and released by CCOC cell lines

**Fig. 3:**
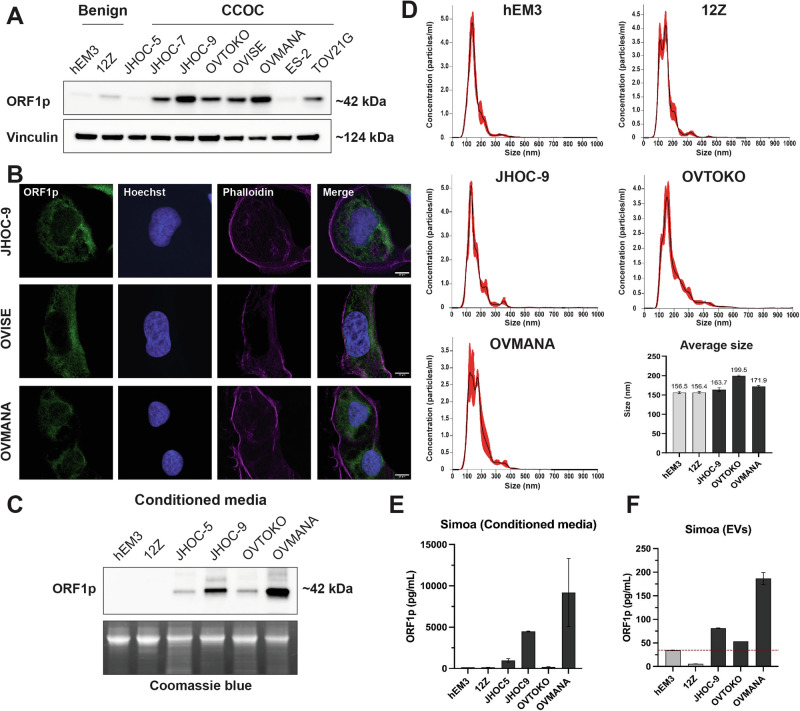
LINE-1 ORF1p is expressed and released by CCOC cell lines. **A** ORF1p expression (WB) in hEM3 (endometrial cell line), 12Z (endometriotic cell line), and a panel of clear cell ovarian carcinoma (CCOC) cell lines. **B** ORF1p expression (green) by immunofluorescence in CCOC. Scale bars: 10 µm. **C** ORF1p detection (WB) in conditioned media from benign and CCOC cell lines. Coomassie blue was used as loading control. **D** Size distribution profile of extracellular vesicles (EVs) characterized via nanoparticle tracking analysis. Bar plot indicates the average size (nm) of EVs in each cell line. Data are shown as mean ± SD. **E** ORF1p detection by Simoa assay in conditioned media and **F** sEVs. Bar plots indicate the average level of ORF1p (pg/mL) in each sample. Data are shown as mean ± SD.

The presence of ORF1p in CCOC precursors prompted us to further investigate ORF1p expression in the development of the disease. Currently, there is a lack of in vitro models for the study of endometrium-related and endometriosis-related malignancies. This makes it challenging to explore disease biology and the mechanisms leading to carcinogenesis. Nevertheless, we obtained one human endometrial epithelial cell line (hEM3)^30^ and one immortalized human endometriotic cell line (12Z)^31^ for evaluating ORF1p levels in culture, along with a panel of eight human CCOC cells. Consistent with the TMA data, western blot analysis showed that ORF1p is readily detectable at variable degrees in the evaluated CCOC cell lines (Fig. 3A). Intracellular ORF1p distribution assessed by immunofluorescence showed a predominantly cytoplasmic distribution of ORF1p, which was observed in IHC images and has been previously established in the literature^3,5,6,13^ (Fig. 3B). As expected, the endometrial cell line hEM3 did not show ORF1p expression, while the endometriotic 12Z cell line showed weak expression. These results support the previous observation that ORF1p expression is acquired in endometriotic lesions and that its expression is retained during the establishment of the carcinoma.

It has been demonstrated that ORF1p can be released by cancer cells^13,26^. To evaluate if ORF1p is a good candidate for detection in extracellular fluids in CCOC, we assessed if cultured cells could release ORF1p into the media. Western blot analysis of cell supernatants showed that the majority of CCOC cell lines had detectable ORF1p in conditioned media and that their levels correlated with those observed in cell lysates (Fig. 3C). To investigate the mechanism underlying ORF1p release, we isolated small extracellular vesicles (sEVs) from conditioned media using size exclusion methodology and assessed the presence of ORF1p. Nanoparticle tracking analysis showed that obtained samples were enriched in small vesicles, ranging from 50 to 250 nm, in all five cell lines (Fig. 3D and Supplementary Fig. 3). To detect ORF1p in these samples, we used a single-molecule array (Simoa) assay, a digital bead-based ELISA technology able to detect low femtomolar ORF1p concentrations^26^. As a control, we first evaluated Simoa on supernatants of hEM3, 12Z, JHOC-5, JHOC-9, OVTOKO, and OVMANA lines, finding the same trend observed by western blot, with no or low ORF1p presence in benign cells and higher levels in CCOC (Fig. 3E). Finally, we found that ORF1p was detectable in sEVs isolated from CCOC cell lines (Fig. 3F), suggesting this as one of the cellular mechanisms by which ORF1p reaches the extracellular media. Together, these data support further exploration of ORF1p as a biomarker for CCOC disease.

### LINE-1 ORF1p is detectable in CCOC patient blood samples

**Fig. 4:**
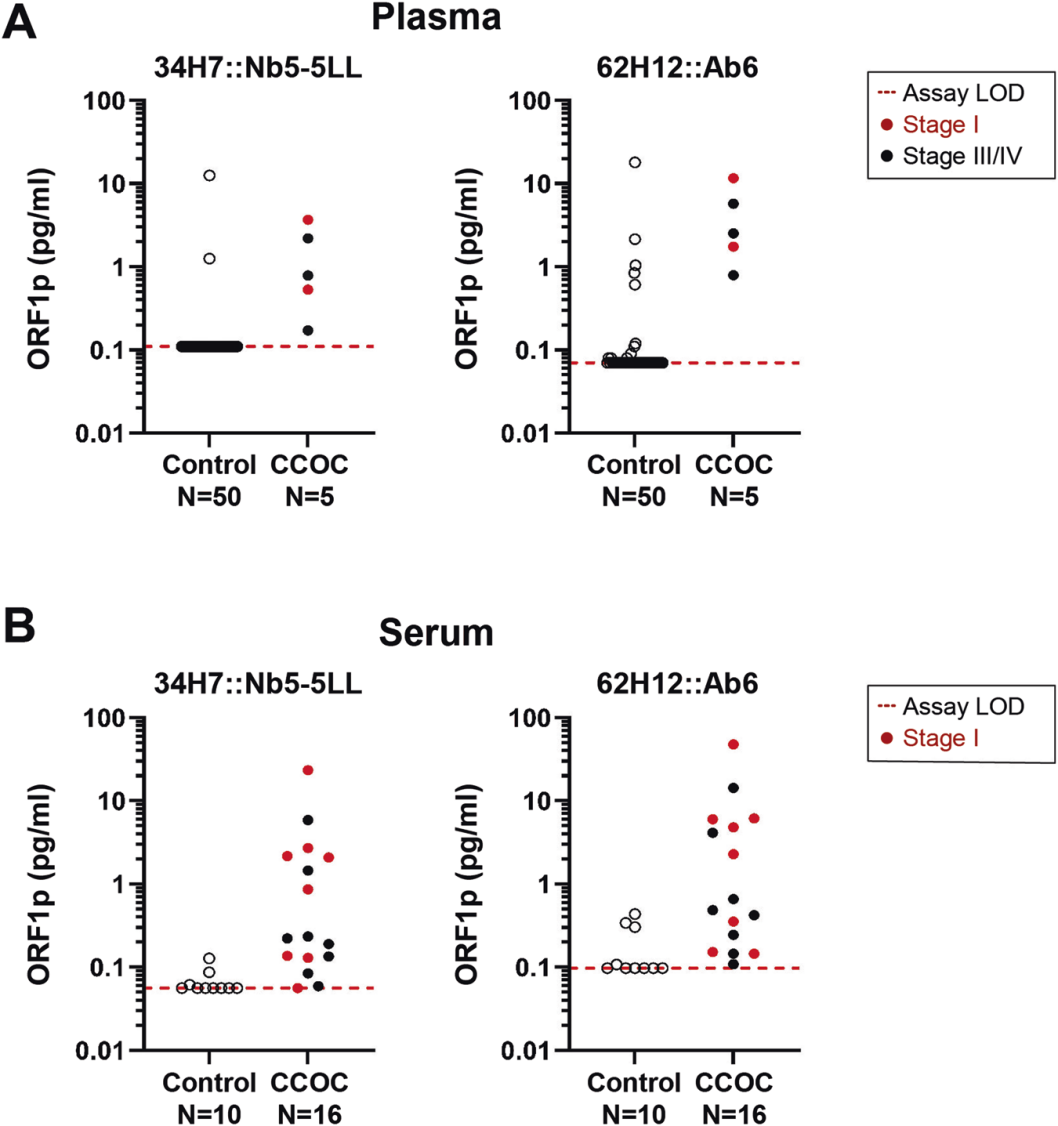
LINE-1 ORF1p is detectable in CCOC patient blood samples. **A** Circulating plasma ORF1p levels detected by two Simoa assays (34H7::Nb5-5LL left and 62H12::Ab6 right) in control (*N* = 50) and CCOC patients (*n* = 5). **B** Circulating serum ORF1p levels detected by two Simoa assays (34H7::Nb5-5LL left and 62H12::Ab6 right) in control (*N* = 10) and CCOC patients (*n* = 16). Patients’ cancer stages depicted when reported. LOD Limit of detection.

We recently showed that LINE-1 ORF1p can be detected in blood samples from patients with HGSOC using both immune-multiple reaction monitoring assays coupled to mass spectrometry (iMRM-MS)^13^ and Simoa^26^. To investigate if this is also true for CCOC, we applied two highly sensitive Simoa assays 34H7::Nb5-5LL and 62H12::Ab6 (written capture::detector), achieving detection limits of 0.016–0.029 pg/mL (106–204 aM trimeric ORF1p), to a small cohort of plasma samples from CCOC patients (*n* = 5). The fundamental sensitivity of these assays remains consistent with previous work^26^, as evidenced by the attomolar-range limits of detection (LOD) for endogenous ORF1p. The approach is conceptually similar to an ELISA but carried out with single molecule sensitivity on a bead: a first antibody captures single molecules of ORF1p on magnetic beads; detection is then achieved using a distinct second, enzyme-conjugated affinity reagent. These assays afford attomolar-range limits of detection (LOD) for endogenous ORF1p^26,32^. As depicted in Fig. 4A, both assays successfully detected ORF1p in five out of five patients (at a specificity 96% for 34H7::Nb5-5LL, and 84% for 62H12::Ab6), including 2 out of 2 presenting with Stage I cancer. To comprehensively characterize ORF1p and further evaluate its reliability as a biomarker, we extended our investigation to serum samples (*n* = 16) from CCOC patients using the same Simoa assays. 34H7::Nb5-5LL detected 7 out of 8 Stage I CCOC cases with a specificity of 80% while 62H12::Ab6 detected all cancer cases including the 8 Stage I (Fig. 4B). Together, our data show promise for ORF1p as an early detection biomarker for CCOC.

### ORF1p regulation in CCOC and precursor cells

**Fig. 5:**
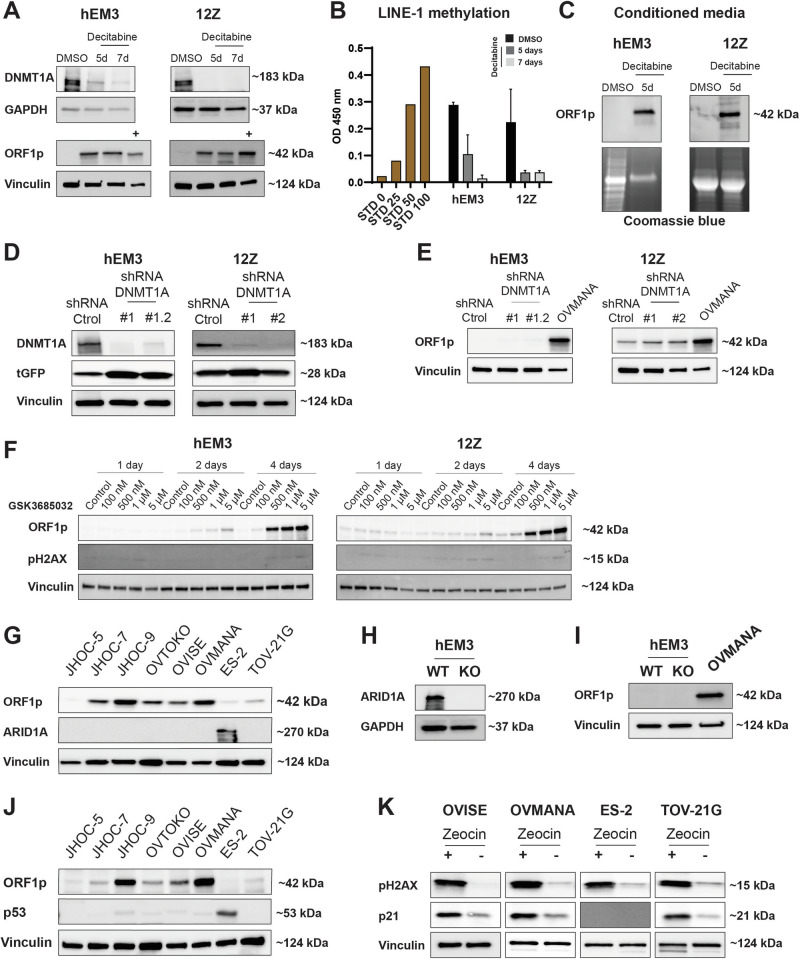
ORF1p expression regulation in CCOC and precursor cells. **A** DNMT1A and ORF1p expression (WB) in hEM3 and 12Z cells after decitabine treatment for 5 or 7 days. DMSO was used as control. OVMANA lysate (+) was used as positive control for ORF1p expression. **B** LINE-1 methylation after decitabine treatment. STD: Standard. Data are shown as mean ± SD. **C** ORF1p expression in conditioned media from hEM3 and 12Z cell lines. Coomassie blue was used as loading control. **D** DNMT1A and **E** ORF1p expression (WB) in hEM3 and 12Z cells transduced with lentiviral shDNMT1A or shRNA control. Turbo-GFP (tGFP) was used as a marker for lentiviral integration. **F** LINE-1 ORF1p and pH2AX levels (WB) after treatment with the DNA methylation inhibitor, GSK3685032, for 1, 2, or 4 days in hEM3 and 12Z cells. **G** ORF1p and ARID1A detection (WB) in a panel of CCOC cell lines. **H** ORF1p and **I** ARID1A detection (WB) in hEM3 WT and KO cells. OVMANA lysate (+) was used as positive control for ORF1p expression. **J** ORF1p and p53 expression (WB) in a panel of CCOC cell lines. **K** pH2AX (marker for DNA damage) and p21 detection in CCOC cell lines after zeocin treatment (200 µg/ml for 16 h). GAPDH and Vinculin were used as loading controls. *n* = 3.

We and others have previously shown that DNA methylation is a major regulator of ORF1p expression in benign epithelial cells^10,12,33^. To explore whether the regulation of ORF1p expression in CCOC and precursors is also regulated by DNA methylation, we treated three CCOC cell lines expressing low levels of ORF1p, JHOC-5, JHOC-7, and ES-2, with the DNMTs inhibitor decitabine (5 µM) for 5 and 7 days. As is shown in Supplementary Fig. 4, decitabine treatment abrogates DNMT1A protein levels, decreases LINE-1 methylation, and induces ORF1p expression in CCOC cells when compared to DMSO-treated condition. Interestingly, when we repeated the treatment using endometrial hEM3 and endometriotic 12Z cells, we observed a clear decrease in LINE-1 methylation and upregulation of ORF1p that was comparable to levels seen in the OVMANA cancer cell line (Fig. 5A, B). Moreover, both hEM3 and 12Z cell lines display robust amounts of ORF1p in their conditioned media after only 5 days of decitabine treatment (Fig. 5C), consistent with our observations in CCOC lines. To better describe the role of DNA methylation in CCOC precursors, and address concerns about decitabine’s pleiotropic effects^34–37^, we constitutively knocked down DNMT1A protein levels in hEM3 and 12Z cells by using shRNAs (shDNMT1A) (Fig. 5D). Interestingly, knockdown of DNMT1A was insufficient to induce ORF1p expression in both benign cells. Compared to OVMANA CCOC line, ORF1p was undetectable in hEM3 treated with either control or DNMT1A shRNA, while no increase was observed in 12Z cells (Fig. 5E). While this may be due to insufficient DNMT1 knockdown, it is worth noting that while decitabine is primary used for its ability to inhibit DNA methylation, it also causes DNA damage^38^. This occurs because decitabine is incorporated into DNA during replication as a nucleoside analog, leading to DNA strand breaks, replication stress, and activation of the DNA damage response. Since DNA damage caused by decitabine can also lead to reactivation of certain genes^39^, we asked whether ORF1p would be expression if we treated cells with a DNMT inhibitor that did not cause DNA damage, specifically GSK3685032. GSK3685032 is a selective DNMT1 inhibitor and acts by binding to the enzyme and preventing it from adding methyl groups to the DNA during replication^38^. Treatment of hEM3 and 12Z cells with GSK3685032 lead to robust ORF1p expression within 4 days with no appreciable DNA damage (Fig. 5F). Similar results were observed in the ES-2 CCOC cell line (Supplementary Fig. 4C). Together, these results indicate that DNA methylation is a major mechanism that regulates ORF1p expression in benign cells and that its loss during neoplastic transformation leads to the reexpression of LINE-1 ORF1p.

Other epigenetic alterations have also been reported to impact LINE-1 de-repression^40^. Given CCOC frequently harbors mutations in the chromatin remodeler gene *ARID1A*^21^, we investigated whether ARID1A might correlate with ORF1p expression levels by examining its status (WT vs. mutated) in our CCOC cohort. *ARID1A* mutations result in the loss of the encoded protein, thus, its status can be assessed through immunohistochemistry^41^. Our analysis revealed that among the patients examined, 22 retained ARID1A staining, suggesting they have the WT *ARID1A* gene, while 18 patients have no ARID1A staining, indicating a mutation in the *ARID1A* gene. However, no correlation between ORF1p levels and ARID1A status was found (Fisher’s Exact test, *p* = 0.74) (Supplementary Fig. 5A). Similarly, there is no clear correlation with ARID1A status in vitro. ES-2, an *ARID1A* WT cell line, and JHOC-5, a cell line with mutations in *ARID1A*, have no ORF1p detectable by western blot. The remaining cell lines, which are *ARID1A* mutated, present variable ORF1p levels (Fig. 5G). Moreover, even knockout of *ARID1A* in endometrial hEM3 cells was insufficient to induce ORF1p when compared to OVMANA levels (Fig. 5H, I).

Although p53 dysregulation is not a common molecular feature of CCOC^42^, the documented association between *TP53* mutations and LINE-1 re-expression and increased LINE-1 retrotransposition^43,44^, prompted us to investigate its role in this context. In our CCOC TMA, only 6 out of 40 cases are *TP53* mutated as per IHC, and those would be predicted to be ORF1p high. Nevertheless, no ORF1p scoring differences were found between *TP53* mutated and wildtype patients (Supplementary Fig. 5B, C), suggesting no correlation between *TP53* mutational status and LINE-1 de-repression. In vitro, ES-2 is the only cell line among the CCOC panel studied that carries a *TP53* mutation, but no ORF1p expression was observed by western blot (Fig. 5J). It is important to note that the absence of mutation does not rule out that p53 may still be functionally impaired in these cell lines. To directly address p53 function, we treated three p53 wildtype cells (OVISE, OVMANA, and TOV21-G) and the p53 mutated ES-2 line with zeocin, a DNA-damaging agent. Zeocin is known to induce double-strand breaks, thereby triggering the activation of DNA damage response pathways. Cells with mutated or compromised p53 are unable to initiate the DNA repair pathway in response to these insults. We found that zeocin induced the p53-p21 repair pathway in all three wildtype cells but not in the p53 mutated line (Fig. 5K), indicating the functional integrity of p53 in CCOC wildtype cell lines.

## Discussion

LINE-1 retrotransposon expression has been demonstrated in numerous human malignancies and precancerous lesions, including those in the gynecologic tract^7,10–15^. Our demonstration of ORF1p expression in CCOC suggests that LINE-1 is induced in the majority (at least 82%) of CCOC. On the contrary, benign endometrium tissue showed little to no expression in the majority of the cases. These findings build on similar results in other organ systems including the gastrointestinal tract and HGSOC, where LINE-1 protein expression is seen in cancers and precursors but not in benign parental epithelium^13,14,26^. One limitation of these studies is the sensitivity and specificity of IHC; it remains unclear whether the weak positivity seen in the cases and in some cycling endometrium represents bona fide low level protein expression or background staining.

The association between endometriosis and subsequent development of CCOC has long been recognized^20,45^. In recent years, this link has been strengthened by the finding of similar recurrent somatic mutations in clear cell carcinomas^21–23,28^, as well as the identification of identical mutations in atypical endometriosis and contiguous CCOC^27,46^. Recent studies have also identified carcinoma-associated mutations in benign endometriosis and even benign endometrial tissue^47–49^. Consequently, CCOC is thought to develop from progression of a subset of endometriotic lesions to atypical endometriosis and eventual carcinoma^21–23,27,28^. In this study, we demonstrated for the first time that ORF1p is expressed in endometriotic precursor lesions. ORF1p expression was found in both typical and atypical endometriotic lesions by IHC, with weak expression in benign endometriosis and increased positivity in atypical lesions, suggesting progressive acquisition of LINE-1 expression (Fig. 2). Findings in cultured cell lines further support this observation, as no ORF1p expression in the endometrial line hEM3 and weak expression in the endometriotic 12Z line was detected (Fig. 3A). Previous studies have demonstrated ORF1p expression in fallopian tube precursors to HGSOC^11,13,15^, but no such finding has been described in precursors to CCOC. The homogeneous expression of ORF1p in endometriotic lesions as well as in carcinomas further supports that LINE-1 dysregulation is an early event in the development of CCOC.

While LINE-1 is implicated as a mobile genetic element in ovarian cancer^50–52^, studies have yet to conclusively established its functional role in ovarian cancer carcinogenesis through insertional mutagenesis. In contrast, in colorectal cancers, the adenomatous polyposis coli (*APC*) tumor suppressor gene can be disrupted by infrequent insertion of LINE-1 elements to drive tumorigenesis in 1-2% of cases^53–55^, but normal colorectal stem cells appear to be a privileged environment tolerating somatic LINE-1 retrotransposition^56^. Across cancers, most LINE-1 retrotransposition insertions occur in non-coding regions, and are likely passenger events rather than a recurrent mediator of tumor suppressor gene loss^3,57–59^. LINE-1 may nonetheless have a key role in carcinogenesis, both serving as a marker of epigenetic dysregulation and perhaps as a mechanism of generation of genome instability and altering cell signaling and the tumor microenvironment; this remains an area of active research^57,58^.

How LINE-1 is de-repressed in cancer cells is actively under investigation. Previous studies have shown that DNA hypomethylation is associated with LINE-1 expression in a variety of tumors, including precursors to HGSOC^11,13,14,16,17^. Consistent with this notion, treatment of benign endometrial (hEM3) and endometriotic (12Z) cells with decitabine, a non-specific DNMT inhibitor, led to expression of ORF1p. Surprisingly, knockdown of DNMT1 did not mimic the effects of decitabine in these cells. Since decitabine is a nucleoside analog of cytosine, it gets incorporated into DNA during replication. This leads to replication stress, DNA breaks, and activation of a DNA damage response^38^. Therefore, to rule out that the effects we were seeing with decitabine were due to DNA damage induced reexpression of ORF1p, we used another DNMT inhibitor that is specific to DNMT1 and does not cause DNA damage (GSK3685032)^38^. Treatment of hEM3 and 12Z cells with GSK3685032 resulted in robust ORF1p expression without DNA damage (Fig. 5F). These results indicate that DNMT1 inhibition is sufficient to cause expression of ORF1p in benign epithelial cells. However, they also suggest that our knockdown of DNMT1 was not able to reduce DNMT1 levels sufficient to see an effect; a finding consistent with the observation that a knockout of DNMT1 in murine cells is embryonic lethal^60^.

To further investigate the mechanisms associated with LINE-1 expression control, we also evaluated if ORF1p de-repression was correlated to *ARID1A* mutations, a common genetic alteration in clear cell ovarian carcinomas, and to *TP53* mutations, a known LINE-1 regulator^43,44^. Assessment of ARID1A and p53 status by immunohistochemistry of our CCOC TMA, revealed no correlation with ORF1p expression. Furthermore, functional studies with *ARID1A* knockout lines and p53 activity, fails to show any dependency of ORF1p expression on these proteins. Together, these findings highlight the complex regulation underlying LINE-1 expression. Additional research is required across various genetic backgrounds to not only elucidate the necessary factors but also to determine the precise sequence and timing of events required for LINE-1 de-repression in CCOC precursor cells.

We demonstrated that tumor cells expressing ORF1p also released it into the extracellular space^59,60^. Our analysis of conditioned media showed this is also true for ORF1p-expressing CCOC lines. The exact mechanism by which cancer cells release ORF1p is not clear, but our studies indicate that sEVs are one mechanism by which ORF1p is release. Our results are consistent with a recent study showing that LINE-1 mRNA and proteins are present in plasma-derived extracellular vesicles from lung cancer patients^61,62^. However, other studies have suggested that the majority of ORF1p resides outside extracellular vesicles and is found in free protein fractions^61^. Despite some differences, these findings together establish that ORF1p can be found in the extracellular space and support its study as a non-invasive cancer biomarker.

Non-invasive cancer biomarkers, including blood markers, are valuable tools for both diagnostic and prognostic purposes. Since binary expression of LINE-1 has been shown in several cancer types, recent efforts have been made to study it as a non-invasive cancer biomarker. By using the liquid biopsy approach, LINE-1 methylation levels have been measured in circulating cell-free DNA from lung and breast cancer samples. Park et al. study found that LINE-1 methylation showed a statistically significant decrease in both cancers compared to healthy controls. In the lung cancer group, the discriminating power of LINE-1 methylation showed an AUC of 0.848 (95% CI: 0.774–0.906), a sensitivity of 75%, and a specificity of 87.50% (cut-off ≤89.65); while in the breast cancer group showed an AUC of 0.890 (95% CI: 0.822–0.938), a sensitivity of 78.12%, and a specificity of 82.81% (cut-off ≤89.86)^63^. Extracellular circulating LINE-1 mRNA in plasma has also been assessed for discriminating among healthy and colorectal cancer patients. In this pilot study by Filipenko et al., LINE-1 mRNA levels were found to be higher in colorectal cancer patients than in healthy controls (*N* = 10)^64^. Given the low LINE-1 ORF1p concentration in blood, the use of standard clinical laboratory techniques for the detection of LINE-1-encoded protein has been more challenging. Recently, we developed immuno-multiple reaction monitoring-mass spectrometry (iMRM-MS) assays to confidently detect extracellular ORF1p in ascites and plasma samples from HGSOC patients. Although we observed a trend for higher ORF1p concentration in cancer patients, the fold change between control and patients did not reach statistical significance (72 cases vs. 37 controls)^26,32^. To overcome sensitivity issues, we developed a series of single molecule ELISA-like immunoassays using single molecule array (Simoa) and flow cytometry (MOSAIC) platforms that improve both sensitivity and specificity of detection as compared to prior other techniques, affording up to low-attomolar-range limits of detection (LOD) for endogenous ORF1p^26,32^. In the present study, we applied the ultrasensitive Simoa approach to assess ORF1p levels in CCOC patients’ plasma (*n* = 5) and serum (*n* = 16) samples. The two Capture::Detection reagent pairs used for the assay were able to detect ORF1p in the patients tested, including samples from early stages. The assessment of these binding pairs underscored the assay’s consistent performance and reproducibility when applied to various biofluids. Although the study did not explicitly compare the performance metrics in serum versus plasma, preliminary data suggest that both matrices enable ORF1p quantification, and the choice depends only on sample availability. Comparative analysis revealed that the 62H12::Ab6 pair achieved higher clinical sensitivity, albeit with compromised specificity, whereas the 34H7::Nb5-5LL pair maintained enhanced specificity with a modest decrease in sensitivity. To maximize diagnostic accuracy and clinical utility, the concurrent application of both assays was recommended, leveraging their complementary sensitivities and specificities. While these approaches need larger cohorts, the biological foundation is well established, and together, our findings support LINE-1 ORF1p further validation as a non-invasive biomarker for CCOC.

## Methods

### Tissue specimens

After institutional review board approval (IRB 702679, The University of Pennsylvania Institutional Review Board, IRB #07), we obtained sections of formalin-fixed, paraffin-embedded (FFPE) human tissue samples to evaluate the expression of ORF1p. Archived tissue samples were drawn from the Departments of Pathology at the Hospital of the University of Pennsylvania (Philadelphia, PA) and the Brigham and Women’s Hospital (Boston, MA), and were originally procured as routine diagnostic surgical specimens. Hematoxylin and eosin (H&E) slides for each case were reviewed by four pathologists (MD, KMD, LES, C P-H) to confirm the presence of cancer or endometriosis. All patients gave written informed consent, and samples were encoded to protect their confidentiality. Our studies have complied with all relevant ethical regulations including the Declaration of Helsinki.

### Blood specimens

Plasma samples used in this study were obtained from the repository of The University of Pennsylvania Ovarian Cancer Research Center, OCRC Tumor BioTrust Collection (IRB 702679, The University of Pennsylvania Institutional Review Board, IRB #07), Research Resource Identifier (RRID): SCR_022387. Serum samples were prospectively collected from women undergoing gynecologic surgery at the University of Chicago Medical Center (U of Chicago IRB 13372B, IRB00000331 #1A BSD/UCMC IRB, IRB00000735 #1B BSD/UCMC IRB, and IRB00002169 #1C BSD/UCMC IRB). All protocols for blood and clinical data collection were approved by the corresponding Institutional Review Board, and all subjects gave written informed consent. Our studies have complied with all relevant ethical regulations including the Declaration of Helsinki.

### TMA construction

A tissue microarray (TMA) of 40 cases of CCOC was assembled using the TMA Master platform at the Brigham and Women’s Hospital TMA Core facility (Boston, MA). Cases were identified by review of all pathology reports between January 2000, and June 2017, in the Hospital of the University of Pennsylvania database that included a diagnosis of “clear cell carcinoma” involving the ovary. A representative FFPE tissue block was chosen for each patient sample based on review of H&E-stained slides by two pathologists (KMD, LES). Three 1.0 mm cores of tumor from each FFPE tissue block were utilized in TMA construction. Benign tissue from ovary, fallopian tube, liver, and kidney (*n* = 5), and cancer tissue from high-grade serous carcinoma, invasive ductal breast carcinoma, endometrial endometrioid carcinoma, and mesothelioma (*n* = 13) was used as controls (2 cores per case).

### Immunohistochemistry

Immunohistochemical staining (IHC) was performed using Envision Plus/Horseradish Peroxidase system (DAKO). FFPE tissue sections from either constructed TMA (CCOC cases) or whole tissue blocks (endometriosis) were de-paraffinized, rehydrated, and incubated in hydrogen peroxide solution for 15 min to block endogenous peroxidase activity. Antigen retrieval was carried out at 122 °C and 15-20 PSI with a pressure cooker in citrate buffer (pH 6.0) for ~40 min. Sections were incubated with primary antibody for 40 min at RT. The secondary antibody was incubated for 30 min at RT, followed by 3,3′-Diaminobenzidine (DAB) for 5 min. All H&E and IHC images were captured with the Panoramic MIDI II (Epredia) digital slide scanner. See antibody details in Supplementary Table 1.

### LINE-1 ORF1p immunohistochemistry scoring

A monoclonal anti-ORF1p antibody (clone 4H1) was utilized to investigate ORF1p expression. ORF1p staining was reviewed and scored by five anatomic pathologists (KMD, MD, CPH, LES, and RD), using the following 4-tiered scale: *No expression* (all cells negative), *Weak* (weak cytoplasmic positivity), *Intermediate* (moderate intensity cytoplasmic staining), or *Strong* (strong, diffuse cytoplasmic staining). Further, we dichotomized *No expression* and *Weak* as “ORF1p negative”, and *Intermediate* and *Strong* were categorized as “ORF1p positive”. Since all cases were reviewed prior to IHC, the scoring pathologists were unblinded to the diagnosis and focused on scoring ORF1p staining. Representative images of ORF1p IHC scoring scale are shown in Fig. 1A.

### Cell lines

TOV21G, ES-2, OVTOKO, and OVMANA cell lines were obtained from the American Tissue Type Collection (Manassas, VA) and as a gift from Dr. Gottfried Konecny (ULCA, Los Angeles, CA). JHOC-5, JHOC-7, and JHOC-9 were purchased from RIKEN BioResource Center (Tsukuba, Ibaraki, Japan). OVISE was a gift from Dr. David Huntsman (The University of British Columbia, Vancouver, BC). EEC12Z (12Z) cell line was generously provided by Dr. Rugang Zhang (MD Anderson Cancer Center) and hEM3 cells by Dr. Tian-Li Wang (Johns Hopkins University). All cells were incubated at 37 °C with 5% CO_2_ and periodically tested to be free of *Mycoplasma* using the Cambrex MycoAlert assay (University of Pennsylvania Perelman School of Medicine Cell Center). Culture growth media can be found in Supplementary Table 2. All cell lines were authenticated using Short Tandem Repeat (STR) profiling (IDEXX, Columbus, MO).

### LINE-1 ORF1p immunofluorescence

Cells were grown overnight on glass coverslips. Cells were fixed with 4% paraformaldehyde in 1X PBS for 20 min at RT. Permeabilization and blocking were done using 3% BSA, goat serum in 1X PBS for 1 h at RT. Primary anti-ORF1p antibody was incubated overnight at 4 °C. The secondary antibody conjugated to Alexa Fluor 488 Dyes (Molecular Probes; Thermo Fisher Scientific), Hoechst (1:10,000), and Alexa Fluor 633 Phalloidin were incubated for 30 min at RT. Finally, cells were analyzed by microscopy using a Zeiss LSM 880 confocal microscope. See antibody details in Supplementary Table 1.

### Protein lysates and western blot

Whole-cell lysates were prepared using RIPA buffer (Thermo Fisher Scientific) and protein content of lysate was quantified using the Pierce BCA kit (Thermo Fisher Scientific). 20–30 μg of proteins were separated by SDS-PAGE before being transferred to a PVDF membrane using the Trans-Blot Turbo system (Bio-Rad). Membranes were incubated with primary antibody overnight at 4 °C. After washing, membranes were incubated with HRP-conjugated secondary antibody for 1 h at RT. Proteins were detected using SuperSignal^TM^ West Substrates (Thermo Fisher Scientific) and visualized with a Chemi-Doc imaging system (Bio-Rad). See antibody details in Supplementary Table 1. All experiments were performed in biological triplicates. All uncropped western blot membranes are provided in Supplementary Figs. 6 and 7.

### Decitabine treatment

Cells were grown to 40% confluence. Then, 5 μM of Decitabine (TOCRIS) was added to the culture media, and cells were treated for 5 or 7 days. The same amount of DMSO was used as control. After treatment, cells were collected by trypsinization, and protein lysates and western blots were performed as described above. All experiments were performed in biological triplicates.

### GSK3685032 treatment

Cells were grown to 70% confluence. Then, 100 nM, 500 nM, 1 μM, or 5 μM of GSK3685032 (Selleckchem) was added to the culture media, and cells were treated for 1, 2, or 4 days. The same amount of PBS was used as control. After treatment, cells were collected by trypsinization, and protein lysates and western blots were performed as described above.

### Zeocin treatment

Cells were grown to 70–80% confluence. Then, 200 μg/mL of Zeocin (Gibco) was added to the culture media and kept for 16 h. The same amount of DMSO was used as control. After treatment, cells were collected by trypsinization, and protein lysates and western blots were performed as described above. All experiments were performed in biological triplicates.

### Lentiviral shRNA

50,000 cells were seeded in 12-well plates. The next day, two short hairpin RNAs (shRNAs) against DNMT1A (n#1 Clone ID: V3SVHS01_7264142, n#2 Clone ID: V3SVHS01_8614601, Horizon Discovery) were inoculated independently (MOI, Multiplicity of Infection 8) into wells and kept overnight. A non-targeting shRNA (VSC10237, Horizon Discovery) was used as control condition. For each shRNA, two wells were used. After 48 h, antibiotic-based selection was performed for 7 days, and cells were allowed to grow for knockdown efficiency testing.

### LINE-1 methylation assay

LINE-1 methylation was assessed using the Global DNA Methylation - LINE-1 Kit (Active Motif) following the manufacturer’s recommendations. Briefly, one μg of genomic DNA (gDNA) was digested with MseI enzyme (10 U/μL) overnight at 37 °C. Then, 100 ng of digested gDNA was hybridized with LINE-1 probe in a thermal cycler. PCR samples were transferred to a streptavidin-coated plate and incubated for 1 h at RT. Next, the 5-methylcytosine monoclonal antibody (1:100 dilution) was incubated for 1 h at RT followed by an HRP-conjugated secondary antibody 1 h incubation. Developing solution was incubated for 3 min until Stop solution addition. Finally, the plate was read at 450 nm. Methylated and non-methylated DNA standard samples were processed in parallel. All reactions were prepared in technical duplicates.

### Conditioned cell culture media

Cells were grown to 80% confluence. Then, cells were rinsed twice with 1X PBS and cultured for 72 h with phenol-free media without FBS. Conditioned cell culture medium was cleared by one centrifugation step at 300 × *g* for 10 min followed by 2000 × *g* for 30 min to remove dead cells and cell debris. Supernatant proteins were concentrated using a Millipore Amicon Ultra-15 centrifugal filter 10 K (Millipore Sigma). Protein content was quantified using the Pierce BCA kit (Thermo Fisher Scientific) and western blots were performed as described above.

### Small extracellular vesicle isolation from conditioned cell culture media

Extracellular vesicles (EVs) were isolated using size exclusion chromatography (SEC). An optimized protocol developed to enrich small EVs (50–250 nm) was followed using IZON qEV1 70 nm column and automatic fraction collector (AFC). Cell lines were grown in EV-depleted FBS media to collect conditioned media as described above. 30 mL conditioned media was concentrated to 1 mL. Then the concentrated conditioned media was centrifuged at 10,000 × *g* to remove large vesicles and run through the SEC column using AFC according to manufacturer recommendation (IZON). Once the 1 mL samples were absorbed in the column, 13 mL 1X PBS was added to the column and eluted volumes were collected. An initial 4 mL of elution volume (buffer volume) was discarded. Then, 5 fractions (F1-F5, 700 µl each) were collected and pooled. These fractions are designated as a purified collection volume which are enriched with small EVs. Pooled fractions (3.5 mL) were further concentrated to around 100–200 µL using a 10 K MWCO Amicon filter (3000 × *g*, 25 min, 4 °C). EVs were characterized for protein content and particle count using Bradford assay and nanoparticle tracking analysis (NTA). EVs were stored at −80 °C until further use.

### Transmission electron microscopy (TEM)

Morphology of isolated EVs was analyzed using TEM. Briefly, freshly thawed 5 µL of EVs were mixed with 20 µL of filtered 1X PBS. Glow-discharge-treated and carbon-film-coated 300-mesh copper grids were floated in 20 µL of diluted EVs solution for 20 min. Following incubation, grids were washed in six successive water droplets and stained in a droplet of 1% uranyl acetate for 5 s. Grids were dried for 15 min, and images were taken using JEOL JEM-1400 TEM.

### Capillary-based western blot

Expression of EV marker protein CD9 and CD81 was analyzed using capillary-based Simple Western assay (Wes, ProteinSimple). EVs at 0.1–0.2 mg/mL concentrations were used for the assay. The 12–230 kDa Separation module with capillary cartridge (ProteinSimple #SM-W004) was used for the separation of proteins and immunodetection, which takes place in a fully automated capillary system. A secondary anti-rabbit module was used for detection following the manufacturer’s protocol. A rabbit monoclonal CD9 (Cell signaling #13174S) and CD81 (Cell signaling #56039S) detection antibodies were used in a 1:50 dilution ratio. Blot images were taken using Compass software version 6.0.0. (ProteinSimple) using High Dynamic Range 4.0 and contrast was manually adjusted for each sample. 1X PBS control was used for each detection antibody as a negative control for non-specific signals.

### Simoa assays

Paramagnetic beads, conjugation and assay buffers and reagents, and Simoa consumables were obtained from Quanterix Corporation. Antibodies used in the first- and second-generation Simoa assays were obtained from Abcam (Ab6, EPR22227-6) and GenScript (34H7, 62H12). Nanobody reagents were generated as previously described^26^. All affinity reagents were buffer exchanged into PBS if not already obtained in PBS. For capture bead preparation, carboxylated paramagnetic 2.7-μm beads (Homebrew Singleplex Beads, Quanterix Corp.) were washed with 400 μL Bead Wash Buffer (Quanterix Corp.) three times, and cold Bead Conjugation Buffer (Quanterix Corp.) two times. The beads were resuspended in 390 μL cold Bead Conjugation Buffer, to which 10 μL of 10 mg/mL 1-ethyl-3-(3-dimethylaminopropyl) carbodiimide hydrochloride (Thermo Fisher Scientific), freshly dissolved in cold Bead Conjugation Buffer, was added. After shaking at 4 °C for 30 min, the beads were washed once with 400 μL cold Bead Conjugation Buffer before resuspending in the capture affinity reagent solution, diluted in Bead Conjugation Buffer to a final volume of 400 μL. The beads were shaken for two hours at 4 °C for affinity reagent conjugation before being washed twice with 400 μL Bead Wash Buffer and blocked in 400 μL Bead Blocking Buffer (Quanterix Corp.) at room temperature for 30 min. Following the blocking step, the beads were washed sequentially with 400 μL Bead Wash Buffer and Bead Diluent (Quanterix Corp.), before being resuspended in Bead Diluent for storage at 4 °C. A Beckman Counter Z Series Particle Counter was used to count the beads. First-generation Simoa assays used 7 × 10^8^ starting beads, 400 μL wash volumes, 10 μL EDC, and 10 g nanobody. Second-generation Simoa assays used 4.2 × 10^8^ starting beads, 300 μL wash volumes, 6 μL EDC, and 40 μg antibody. For biotinylation of detector reagents, freshly dissolved sulfo-NHS-LC-LC-biotin was added to a 1 mg/mL solution of antibody or nanobody at an 80-fold molar excess. After incubation of the biotinylation reaction at room temperature for 30 min, the reaction was purified with an Amicon Ultra-0.5 mL centrifugal filter (50 K MWCO cutoff for antibody in the first-generation assay; 10 K MWCO for dimeric nanobody in the second-generation assay). Five wash cycles with 1x PBS were carried out at 14,000 × *g* for five minutes, followed by collection of the purified biotinylated detector reagent via inversion of the filter into a new tube and centrifuging at 1000 × *g* for two minutes.

Simoa assays were run on an HD-X Analyzer (Quanterix Corp), following manufacturer instructions for assay reagent loading. Each assay utilized 250,000 capture beads and 250,000 helper (non-conjugated) beads in a three-step assay configuration (15-min target capture from 100 µL sample, 5-minute detector reagent incubation, 5-minute streptavidin-*β*-galactosidase incubation). All plasma samples were diluted four-fold in Homebrew Sample Diluent (Quanterix Corp.) with added 1x Halt Protease Inhibitor Cocktail (Thermo Fisher Scientific). The second-generation Simoa assays contained an additional 1% Triton-X 100 in the Sample Diluent. Concentrations of 0.3 μg/mL detector reagent were used for all assays, and 150 pM and 300 pM streptavidin-*β*-galactosidase were used for the first- and second-generation Simoa assays, respectively. Wash steps between each step were performed with System Wash Buffer 1 (Quanterix Corp). After the final wash, beads were loaded along with the fluorogenic enzyme substrate resorufin *β*-D-galactopyranoside into a 216,000-microwell array. The wells were then sealed with oil, and imaging and counting of “on” and “off” wells were performed by the instrument. The average enzyme per bead (AEB) for each sample was also calculated by the instrument. Calibration curves were fit using a 4PL fit with a 1/y^2^ weighting factor. The assay limit of detection (LOD) was calculated as three standard deviations above the blank.

## Supplementary information

Supplemental Data

## Data Availability

All data generated or analyzed during this study are included in this published article [and its supplementary information files].
