# Supplementary material for: LINE-1 ORF1p expression occurs in clear cell ovarian carcinoma precursors and is a candidate blood biomarker
Source: NPJ Precis Oncol. 2025 Mar 6;9:62. doi: 10.1038/s41698-025-00849-1 (PMC11885553; doi:10.1038/s41698-025-00849-1)
Supplement: Supplementary file 1 — Supplemental Data [file 41698_2025_849_MOESM1_ESM.docx]

**Supplementary Data**

**LINE-1 ORF1p expression occurs in clear cell ovarian carcinoma precursors and is a candidate blood biomarker**

Pamela R. de Santiago, Sho Sato, Stephanie J. Zhang, Meaghan C. Dougher, Kyle M. Devins, Agnes J. Bilecz, Sagar Rayamajhi, Gabriel Mingo, Hannah S. Rendulich, Yi Feng, Connie Wu, Martin S. Taylor, Yelena Zhuravlev, Euihye Jung, Dalia K. Omran, Tian-Li Wang, Ie-Ming Shih, Lauren E. Schwartz, Sarah Kim, Mark A. Morgan, Janos L. Tanyi, Kathleen H. Burns, Ernst Lengyel, Carlos Parra-Herran, Andrew K. Godwin, David R. Walt, Ronny Drapkin.

**Supplementary Figures**


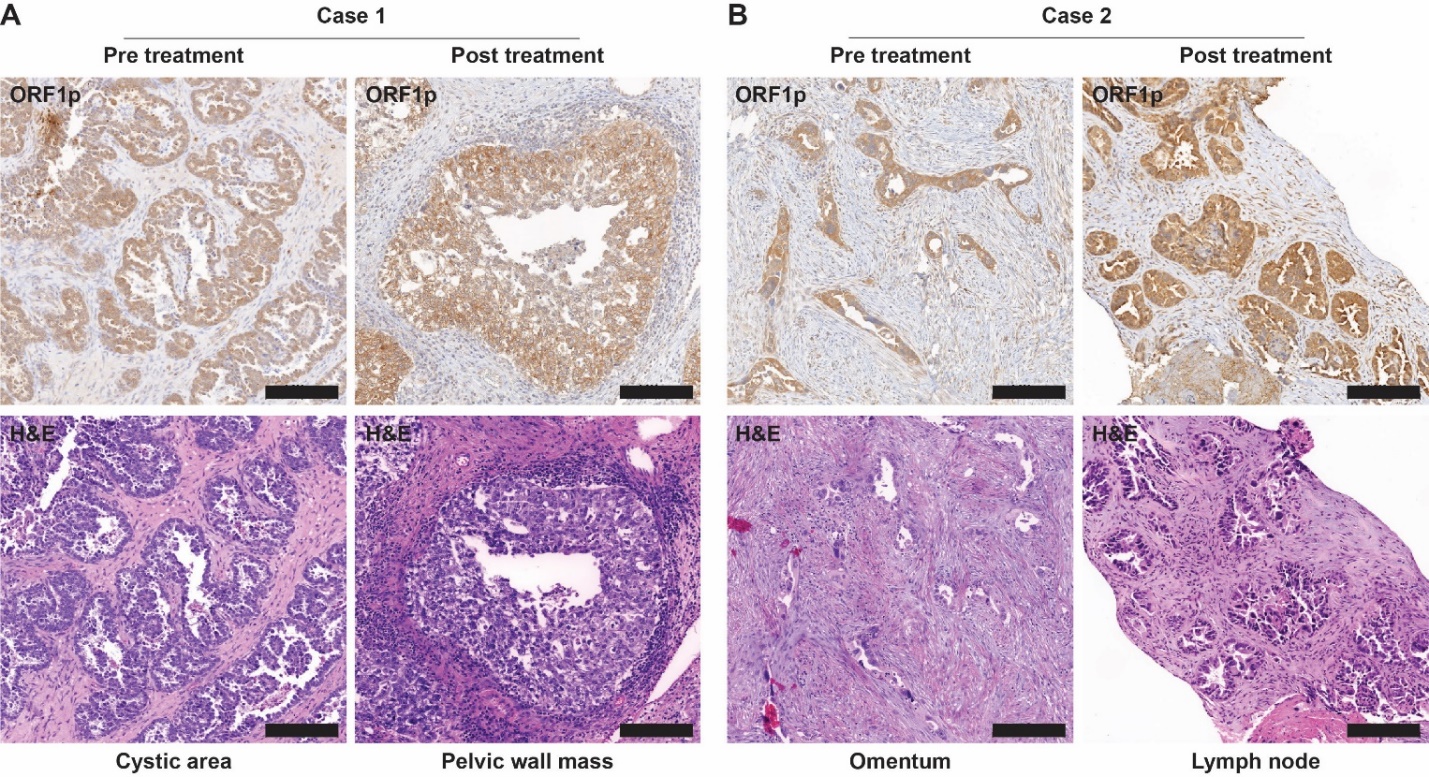


**Supplementary Figure 1. Recurrent clear cell ovarian cancer cases.** **(A and B)** Representative images of ORF1p expression (IHC) (upper panel) and hematoxylin and eosin (H&E) staining (bottom panel) of pre- and post-treatment samples from two cases of recurrent clear cell ovarian carcinoma. Scale bar: 0.200 mm.


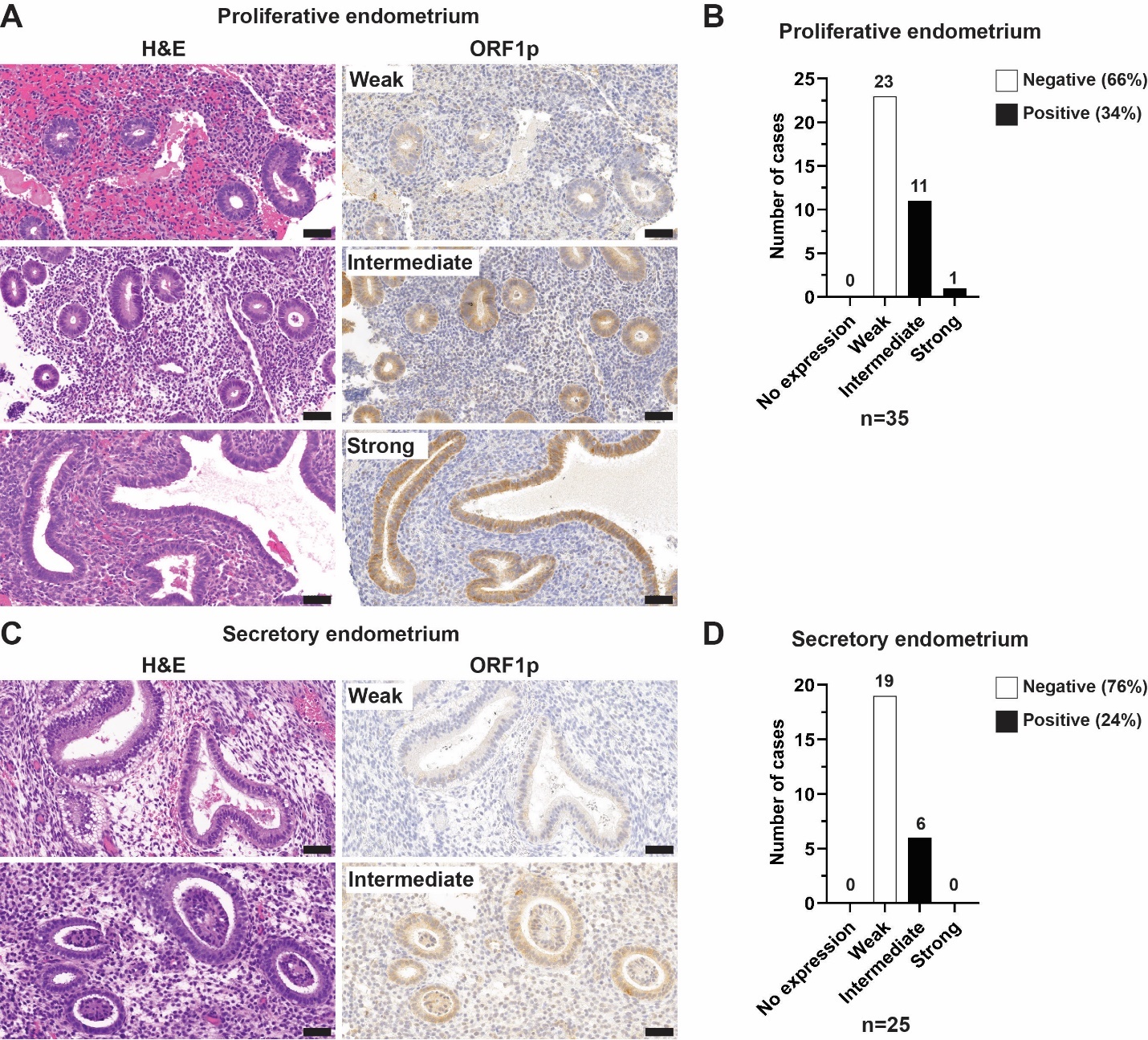


**Supplementary Figure 2.** **LINE-1 ORF1p expression in proliferative and secretory endometrium. (A)** Representative images of hematoxylin and eosin (H&E) staining (left panel) and ORF1p expression (IHC) (right panel) on whole-mount slides from cases of proliferative endometrium. **(B)** ORF1p IHC scoring for proliferative endometrium (n=35). **(C)** Representative images of H&E staining (left panel) and ORF1p expression (IHC) (right panel) on whole-mount slides from cases of proliferative endometrium. **(D)** ORF1p IHC scoring for secretory endometrium (n=25). Scale bar: 0.050 mm.


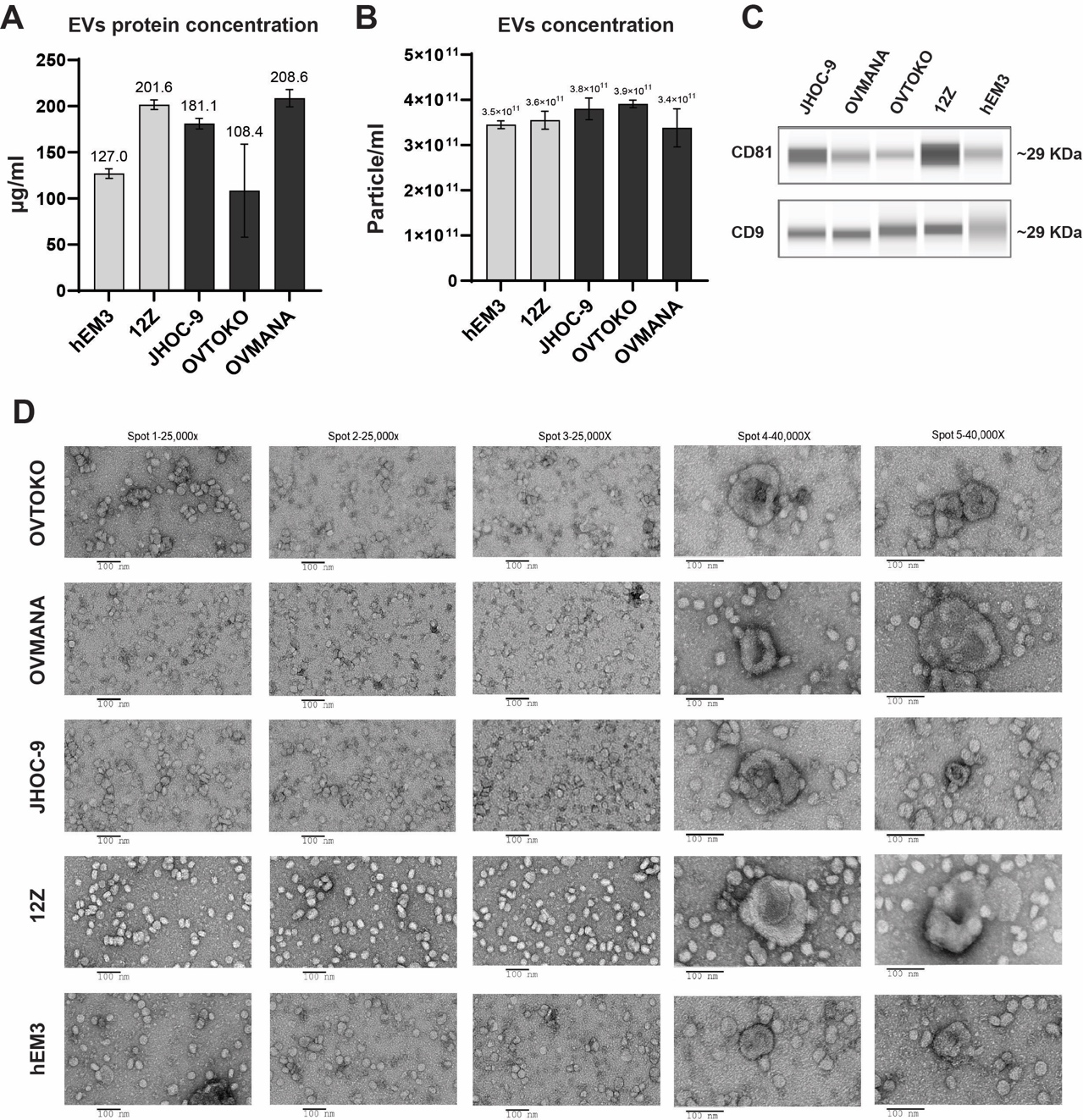


**Supplementary Figure 3.** **Extracellular vesicle isolation validation in hEM3, 12Z, JHOC-9, OVMANA, and OVTOKO cell lines** **(A)** EVs protein concentration measured by Bradford assay. (B) EVs concentration measured by nanoparticle tracking analysis (NTA). **(C)** Capillary-based western blot of EV-associated proteins CD9 and CD81. EVs were isolated from five cell lines and analyzed for the presence of transmembrane proteins CD9 and CD81, considered EV marker proteins. 100-200 µg/mL of EVs were used for the assay. All EVs showed the presence of transmembrane proteins. **(D)** Representative images of EVs via transmission electron microscopy (TEM). TEM shows mostly round shape morphology (spot 1-3) and occasional cup-shaped morphology (spot 4-5). Images shown are five different spots in the TEM grid with 25,000x and 40,000x magnification. In general, EVs less than 100 nm show round-shape morphology while EVs larger than 100 nm show cup-shaped morphology. Scale bar represents 100 nm. Note that majority of EVs shown are below 100 nm in size, which is less than the size reported by NTA (150-200 nm). NTA measures the hydrodynamics radius of EVs which are larger than the geometric size of EVs. In addition, EVs are dehydrated during the TEM staining process, resulting in “shrunken” EVs, further reducing the size of EVs reported by TEM.


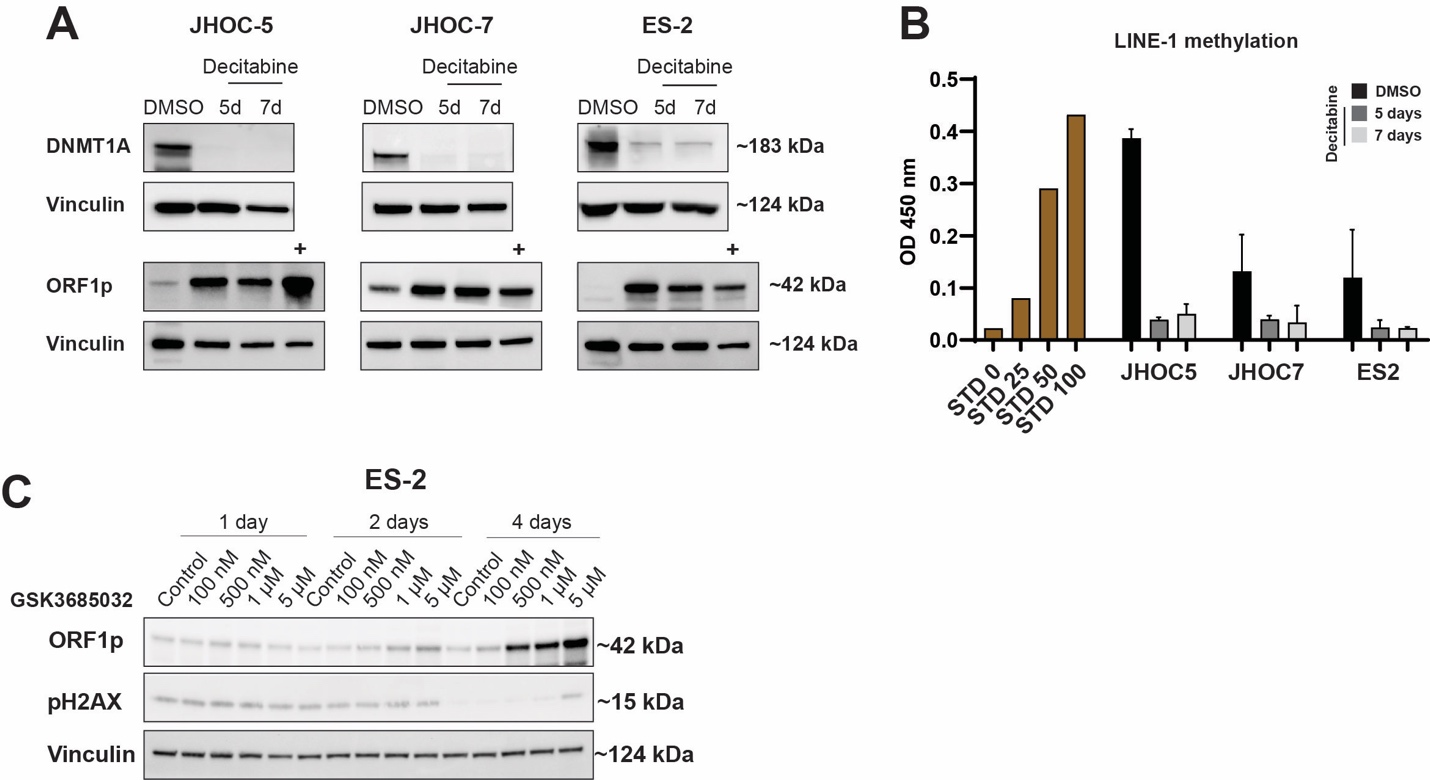


**Supplementary Figure 4. Demethylating treatment of CCOC cell lines leads to LINE-1 de-repression. (A)** DNMT1A and LINE-1 ORF1p expression after decitabine treatment of JHOC-5, JHOC-7, and ES-2 cell lines. OVMANA lysate (+) was used as positive control for ORF1p expression. (n=3). **(B)** LINE-1 methylation across standards (STD) and CCOC cell lines after decitabine treatment. DMSO was used as control condition. (n=3). **(C)** LINE-1 ORF1p and pH2AX levels after treatment with the DNA methylation inhibitor, GSK3685032, for 1, 2, or 4 days in ES-2 cells. Vinculin serves as loading control.


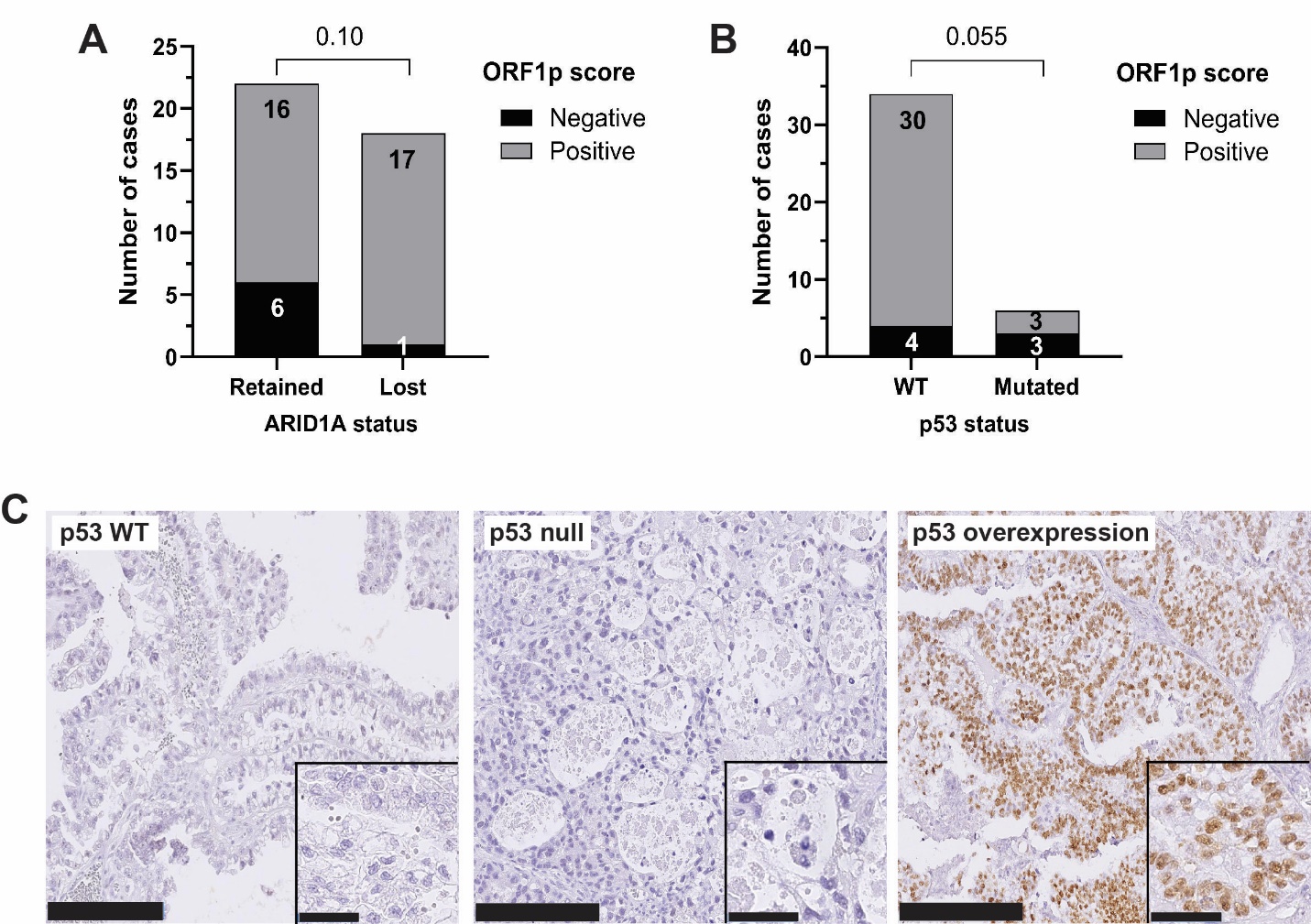


**Supplementary Figure 5.** **ARID1A and p53 correlation with LINE-1 ORF1p expression in the CCOC patient’s cohort. (A)** ARID1A and **(B)** p53 mutational status by IHC and its correlation with ORF1p staining. *p*-value by Fisher’s Exact Test. n=40. **(C)** Representative IHC images of p53 wildtype and mutated (null and overexpression) cases. Scale bar: 250 µm.

**
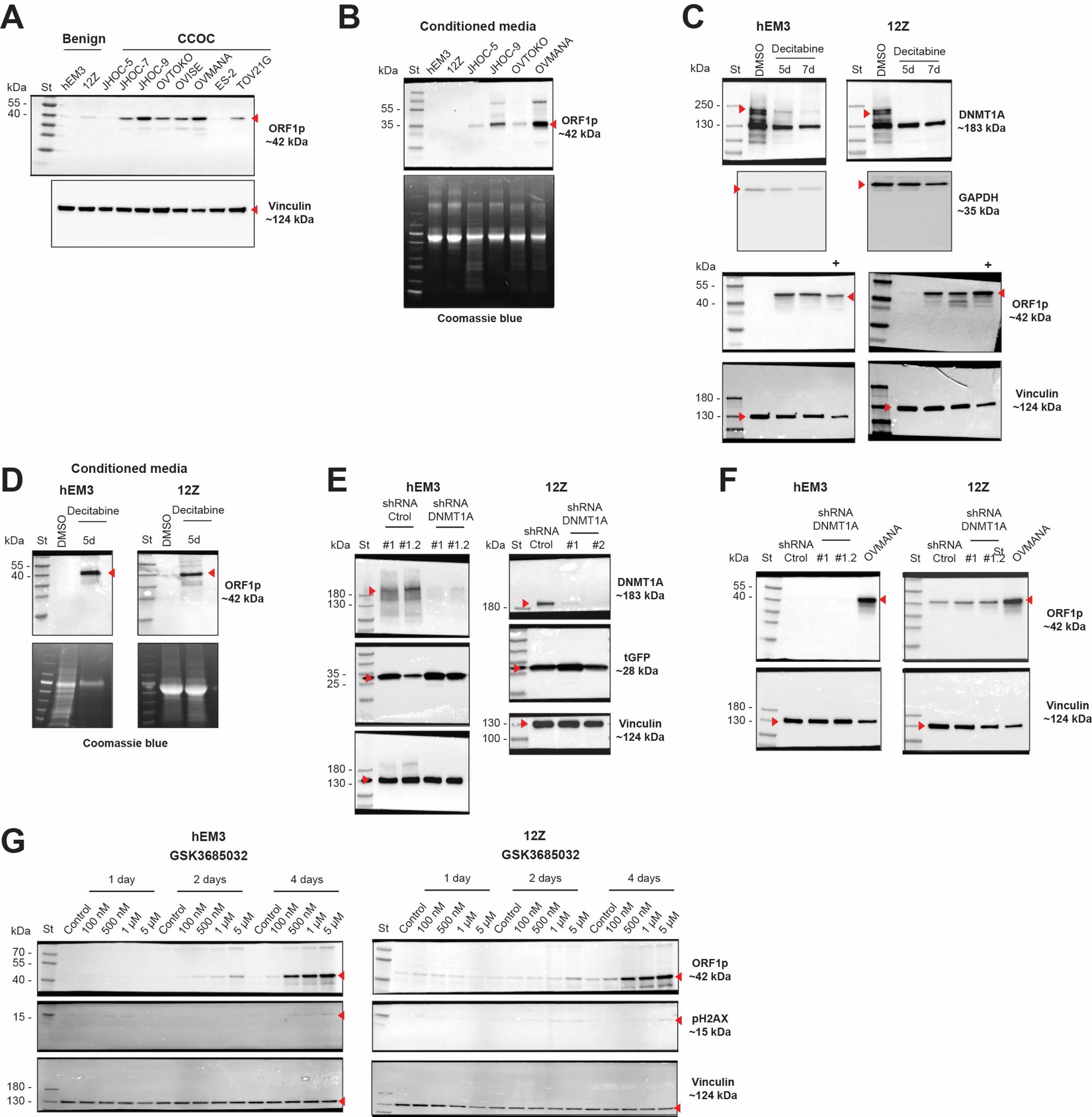
**

**Supplementary Figure 6. Uncropped western blots for** **(A)** Figure 3A, **(B)** Figure 3C, **(C)** Figure 5A, **(D)** Figure 5C, **(E)** Figure 5D, **(F)** Figure 5E, and **(G)** Figure 5F. The standards (St) for molecular weight used were PageRuler™ Plus Prestained Protein Ladder, 10 to 250 kDa (Thermo Fisher Cat. #26620), or PageRuler™ Prestained Protein Ladder, 10 to 180 kDa (Thermo Fisher Cat. #26617).

**
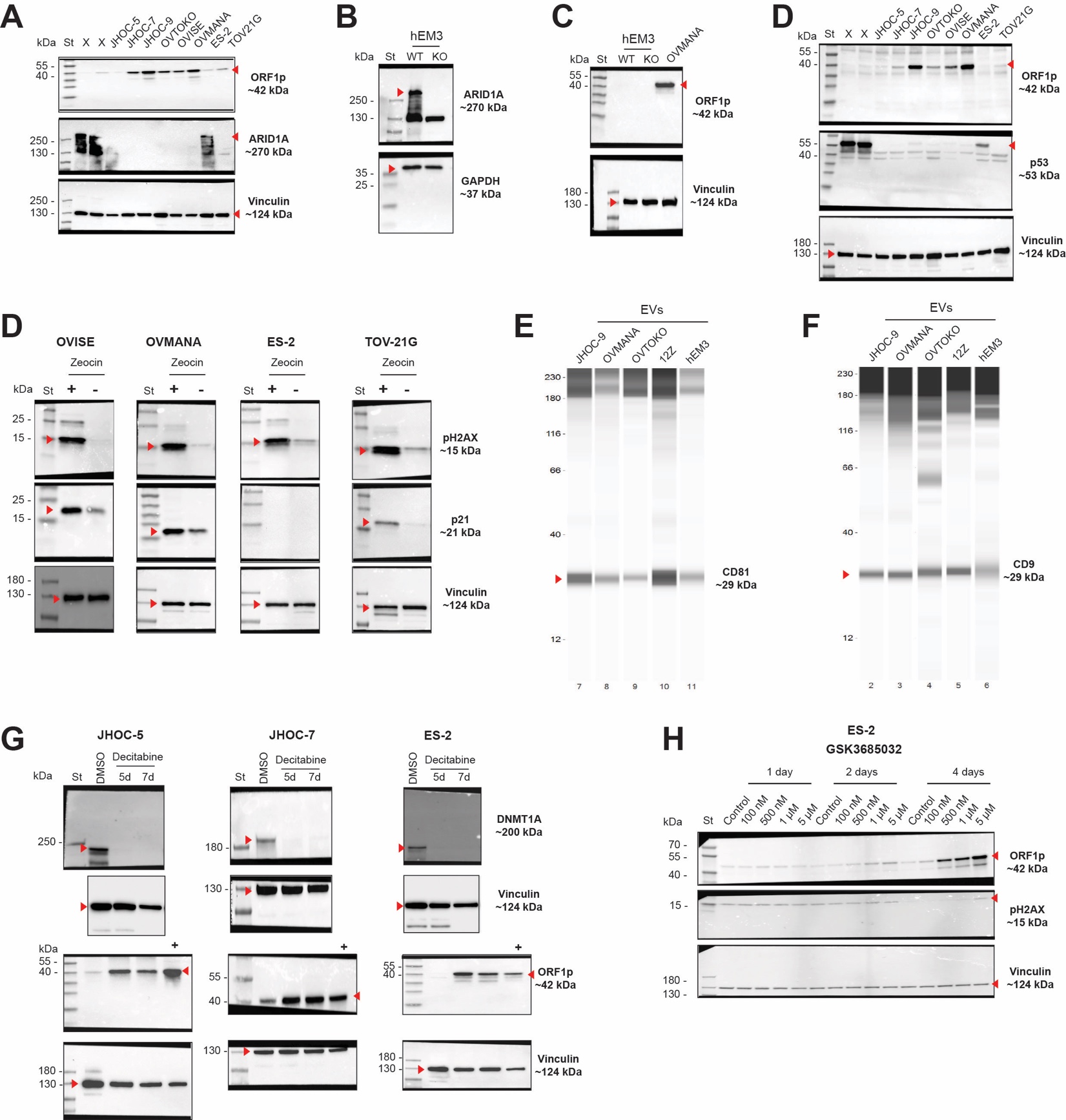
**

**Supplementary Figure 7. Uncropped western blots for (A)** Figure 5G, **(B)** Figure 5H, **(C)** Figure 5I, **(D)** Figure 5J, **(E)** Figure 5K, **(F)** Supplementary Figure 3C, **(G)** Supplementary Figure 4A, and **(H)** Supplementary Figure 4C. The standards (St) for molecular weight used were PageRuler™ Plus Prestained Protein Ladder, 10 to 250 kDa (Thermo Fisher Cat. #26620), or PageRuler™ Prestained Protein Ladder, 10 to 180 kDa (Thermo Fisher Cat. #26617).

**Supplementary Tables**

**Supplementary Table 1.** Antibodies and dilutions used in this study.

| **Antibody** | **Vendor** | **Catalog #** | **Dilution** |
| --- | --- | --- | --- |
| **Immunohistochemistry** | | | |
| ORF1p | Sigma-Aldrich | MABC1152 | 1:300 |
| **Western blot** | | | |
| ORF1p | Sigma-Aldrich | MABC1152 | 1:1000 |
| Vinculin | Cell Signaling | 13901 | 1:1000 |
| DNMT1A | Cell Signaling | 5032 | 1:1000 |
| p53 | Cell Signaling | 9282 | 1:1000 |
| ARID1A | Cell Signaling | 12354 | 1:1000 |
| Turbo-GFP | Invitrogen | PA5-22688 | 1:1000 |
| GAPDH | Cell Signaling | 2118 | 1:1000 |
| p21 | Invitrogen | MA5-31479 | 1:1000 |
| pH2AX | Cell Signaling | 2577 | 1:1000 |
| **Immunofluorescence** | | | |
| ORF1p | Sigma-Aldrich | MABC1152 | 1:300 |
|  |  |  |  |

**Supplementary Table 2.** Cell lines and growth media used in this study.

| **Cell line** | **Culture media** |
| --- | --- |
| hEM3 | DMEM/F12 + 10% FBS + 1% Pen/Strep |
| EEC12Z (12Z) | DMEM/F12 + 10% FBS + 1% Pen/Strep |
| JHOC-5 | DMEM/F12 + 10% FBS + 0.1mM Non-Essential Amino Acids + 1% Pen/Strep |
| JHOC-7 | DMEM/F12 + 10% FBS + 0.1mM Non-Essential Amino Acids + 1% Pen/Strep |
| JHOC-9 | DMEM/F12 + 10% FBS + 0.1mM Non-Essential Amino Acids + 1% Pen/Strep |
| OVTOKO | RPMI-1640 + 10% FBS + 1% Pen/Strep |
| OVISE | RPMI-1640 + 10% FBS + 1% Pen/Strep |
| OVMANA | RPMI-1640 + 10% FBS + 1% Pen/Strep |
| ES-2 | McCoy’s 5A + 10% FBS + 1% Pen/Strep |
| TOV-21G | MCDB 105: Medium 199 + 10% FBS + 1% Pen/Strep |

**Supplementary Table 3. Correlation between ORF1p scores and clinicopathological characteristics in the CCOC cohort.**

|  | **ORF1p score** | | ***p*-value** |
| --- | --- | --- | --- |
|  | **Negative** | **Positive** |  |
| **Age at diagnosis** | | | |
| Mean  (St Dev) | 60.62 (11.78) | 57.04  (11.30) | 0.55 |
| **FIGO Stage** | | | |
| Early Stage (I & II) | 4 | 3 | >0.99 |
| Advanced Stage (III & IV) | 14 | 12 |  |
| **Tumor size** | | | |
| Mean  (St Dev) | 10.07  (4.797) | 12.63  (6.767) | 0.35 |

Age at diagnosis and Tumor size were evaluated by using *t*-test. FIGO Stage was evaluated by Fisher's exact test.

**Supplementary Table 4. ORF1p scores in typical and atypical endometriosis**

| **Endometriosis** | **ORF1p score** | | **Total**  **cases** | ***p*-value** |
| --- | --- | --- | --- | --- |
|  | **Negative** | **Positive** |  |  |
| Typical endometriosis | 43 | 19 | 62 | 0.0001 |
| Atypical endometriosis | 12 | 28 | 40 |  |

*p*-value by Fisher's exact test
